# Patients with psychotic disorders exhibit different audio-visual perceptual decision biases and metacognitive abilities

**DOI:** 10.1192/j.eurpsy.2023.334

**Published:** 2023-07-19

**Authors:** L. Franzen, S. Eickhoff, H. Schewe, L. M. Schmitt, J. Erb, S. Borgwardt, C. Andreou, J. Obleser

**Affiliations:** 1 Psychology; 2 Psychiatry and Psychotherapy, University of Lübeck, Lübeck, Germany; 3 Donders Institute for Brain, Cognition and Behaviour, Radboud University, Nijmegen, Netherlands

## Abstract

**Introduction:**

In the inherently noisy real world, we can rarely have full certainty about what we have just seen or heard. Thus, making a perceptual decision on sensory information, and simultaneously tracking our varying levels of certainty in these decisions (i.e., metacognitive abilities) are crucial components of everyday life.

Hallucinations, such as confidently reporting a human voice or face when none was present, are a hallmark of psychotic disorders but also occur among the normal population. Particularly in patients with psychotic disorders, these misperceptions are linked to confident beliefs in their actual existence. However, whether patients’ confidence is only increased during such erroneous perceptions and whether perceptual and metacognitive decisions arise from supramodal mechanisms across sensory modalities remains unknown.

**Objectives:**

In the laboratory, we tested perceptual and metacognitive decisions under varying levels of sensory certainty in healthy adults and patients with psychotic disorders admitted to a psychiatry ward (N_con_=32, N_pat_=12; age = 19-49; F2x.x diagnoses).

**Methods:**

Specifically, participants had to detect human voices or faces against briefly presented noisy backdrops and subsequently rate their confidence in the accuracy of their perceptual decision (Fig 1A,B,C). We further hypothesised that probabilistic cues prior to blocks of trials can bias participants’ choices and hallucination probability (i.e., confident false alarms).

**Results:**

Patients exhibited higher perceptual sensitivity in the auditory than the visual task, alongside a generally stronger decision bias towards fewer ‘voice/face’ choices (Fig 2A,B). This bias was more pronounced in the visual domain. Decision performance was overall higher on the auditory task but lower for patients (predicted minimum > 55%; Fig 2C). Strong correlations between auditory accuracy and PANSS hallucination scores of patients and LSHS scores of healthy participants suggest an effect of these hallucinatory experiences on accurate perception.

Metacognitive abilities were reduced in patients across both modalities: They exhibited general overconfidence, which was stronger for incorrect trials (Fig 3A). Patients’ confidence ratings were inversely related to the probability of choosing ‘voice/face’. Combining both perceptual and confidence decisions, patients showed higher hallucinations probability in the auditory task, particularly in more difficult trials (i.e., with less informative sensory evidence; Fig 3B).

**Image:**

**Figure:**
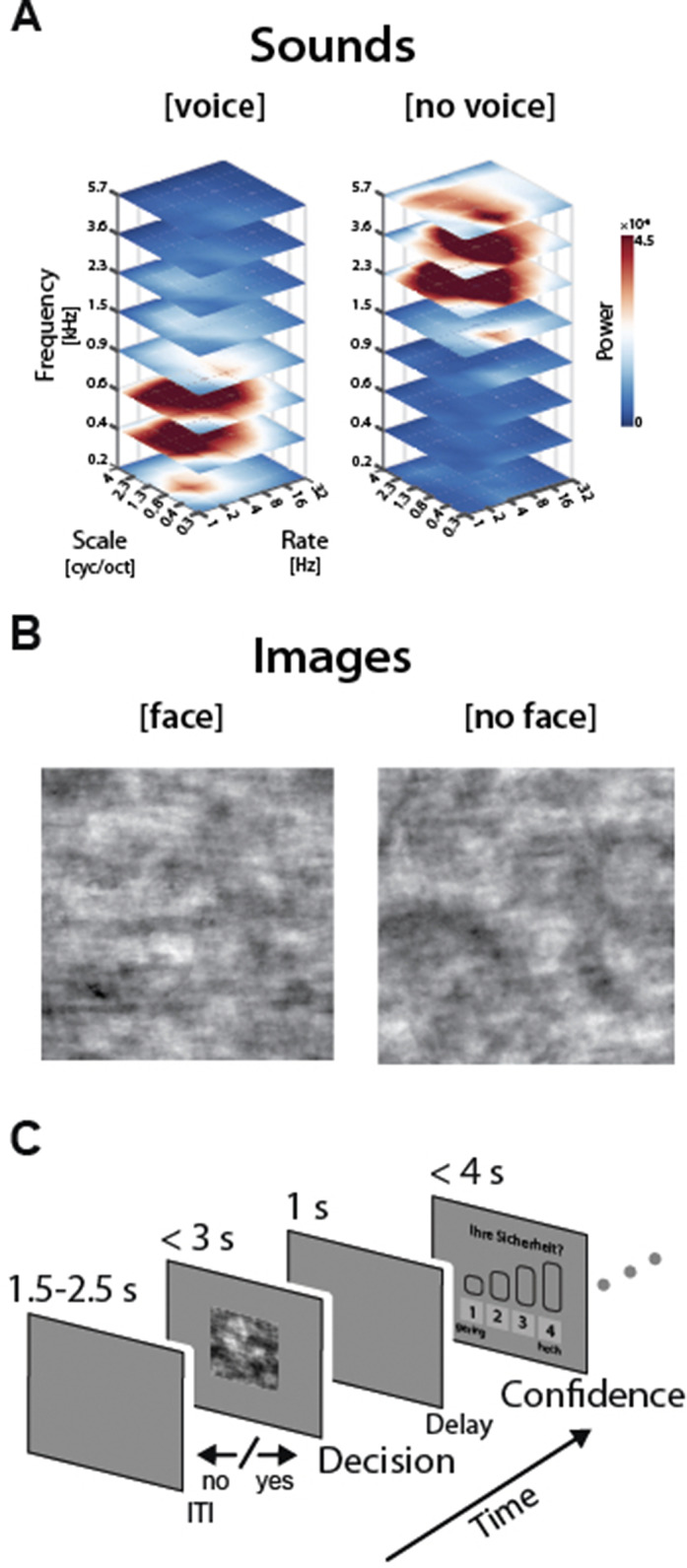

**Image 2:**

**Figure:**
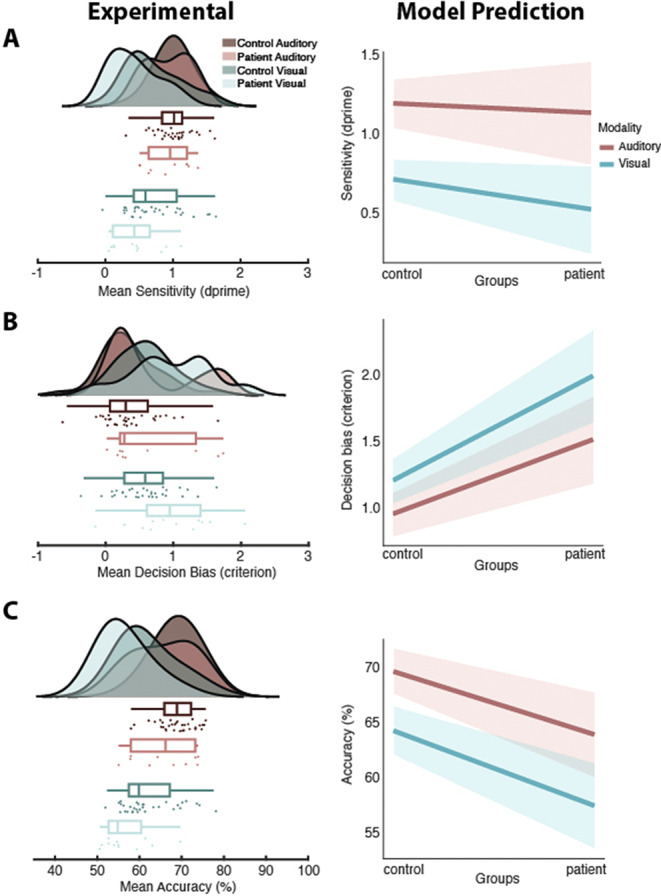

**Image 3:**

**Figure:**
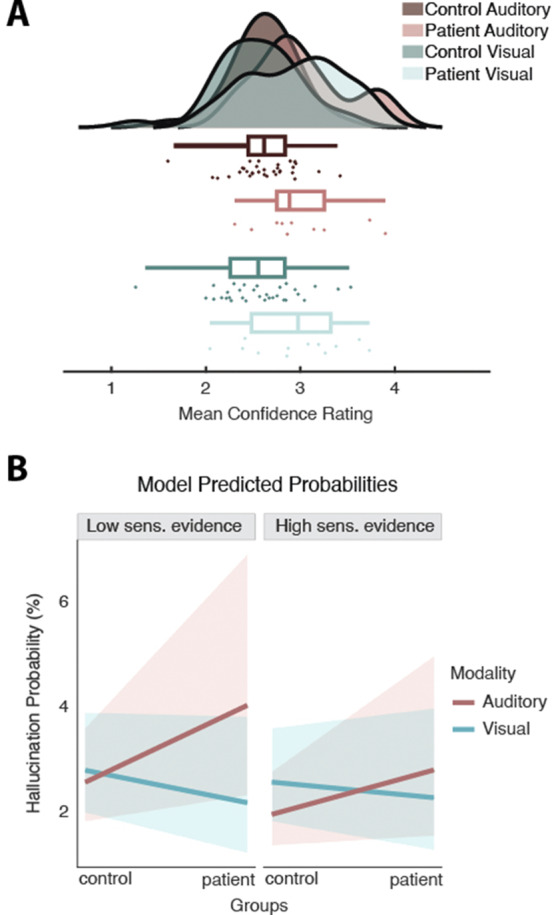

**Conclusions:**

In sum, patients with psychotic disorders exhibit increased decision bias accompanied by increased confidence, and thus a reduced fidelity in their metacognitive abilities. The modality differences are in line with phenomenology and reported hallucination rates. These results suggest stronger priors in psychotic disorders resulting in worse perceptual acuity and assessment of this perception.

**Disclosure of Interest:**

None Declared

